# Production of Polyunsaturated Fatty Acids and Lipids from Autotrophic, Mixotrophic and Heterotrophic cultivation of *Galdieria* sp. strain USBA-GBX-832

**DOI:** 10.1038/s41598-019-46645-3

**Published:** 2019-07-25

**Authors:** Gina López, Camilo Yate, Freddy A. Ramos, Mónica P. Cala, Silvia Restrepo, Sandra Baena

**Affiliations:** 1 Unidad de Saneamiento y Biotecnología Ambiental (USBA), Departamento de Biología, Pontificia Universidad Javeriana, POB 56710 Bogotá DC, Colombia; 2 Facultad de estadística, Universidad Santo Tomas, Carrera 9 # 51-11, Bogotá DC, Colombia; 3 Universidad Nacional de Colombia-Sede Bogotá, Facultad de Ciencias, Departamento de Química, Carrera 30 # 45-03, Bogotá DC, Colombia; 4 Department of Chemistry, Universidad de los Andes, Cra 1 No. 18A-12, Bogotá DC, Colombia; 5 Biological Sciences Department, Universidad de los Andes, Cra 1 No. 18A-12, Bogotá DC, Colombia; 6 Present Address: Unidad de Saneamiento y Biotecnología Ambiental (USBA), Departamento de Biología, Pontificia Universidad Javeriana, POB 56710 Bogotá DC, Colombia

**Keywords:** Microbiology, Environmental microbiology

## Abstract

A search for extremophile organisms producing bioactive compounds led us to isolate a microalga identified as *Galdieria* sp. USBA-GBX-832 from acidic thermal springs. We have cultured *Galdieria* sp. USBA-GBX-832 under autotrophic, mixotrophic and heterotrophic conditions and determined variations of its production of biomass, lipids and PUFAs. Greatest biomass and PUFA production occurred under mixotrophic and heterotrophic conditions, but the highest concentration of lipids occurred under autotrophic conditions. Effects of variations of carbon sources and temperature on biomass and lipid production were evaluated and factorial experiments were used to analyze the effects of substrate concentration, temperature, pH, and organic and inorganic nitrogen on biomass production, lipids and PUFAs. Production of biomass and lipids was significantly dependent on temperature and substrate concentration. Greatest accumulation of PUFAs occurred at the lowest temperature tested. PUFA profiles showed trace concentrations of arachidonic acid (C_20:4_) and eicosapentaenoic acid (C_20:5_). This is the first time synthesis of these acids has been reported in *Galdieria*. These findings demonstrate that under heterotrophic conditions this microalga’s lipid profile is significantly different from those observed in other species of this genus which indicates that the culture conditions evaluated are key determinants of these organisms’ responses to stress conditions and accumulation of these metabolites.

## Introduction

Production of complex, high-added-value molecules from microalgae has taken off in recent years^1–3^. These molecules include proteins, pigments and vitamins, but lipids, particularly polyunsaturated fatty acids (PUFAs), are of particular interest because of their many applications in cosmeceuticals, nutraceuticals, fine chemicals, pharmaceutics, and biodiesel^4,5^.

Most lipids are composed primarily of glycerol and highly unsaturated fatty acids with chains of twelve or more carbon atoms^5^. Fatty acids produced by microalgae that have carbon chains between C_14_ and C_20_ are usually used to produce biodiesel^6^. Lipids produced by microalgae that have chains longer than C_20_ are mostly PUFAs which include ω-6 and ω-3 fatty acids such as docosahexaenoic acid (DHA) and eicosapentaenoic acid (EPA). These are essential nutrients for humans and therefore can be used as food supplements^7–9^. Importantly, PUFAs have demonstrated protective and curative activities against chronic inflammatory diseases as well as properties that are or may be useful for treating cancer, diabetes, atherogenesis, and Alzheimer’s disease^9–12^.

Although lipids fulfill various functions in cell membranes and organelles, lipid reserves are also a form of energy storage that is necessary for cellular metabolism^5^. The average lipid content of microalgae varies from 1% to 70% (w/w) and depends on a cell’s species, life cycle and on the nutritional and environmental requirements of the microalgae^13^. In the laboratory these requirements translate into culture conditions such as composition of the medium, temperature, light intensity, the light/dark ratio used, and the aeration rate^14^. In some species, lipid content can be close to 85% of a cell’s dry weight^15^.

Development of any metabolic engineering strategy requires identification of a pathway used by the microalgae^8^, and two PUFA synthesis pathways have been identified. On the aerobic pathway when molecular oxygen is present, saturated fatty acids react with desaturase and elongase enzymes which results in formation of PUFAs. On the anaerobic pathway, *de novo* synthesis of PUFAs for desaturases occurs through biosynthesis by polyketide synthase (PKS)^6,16^.

Thermoacidophilic microalgae of the order Cyanidiales^17,18^ are the only eukaryotes capable of living in environments with pH lower than 3.0 and temperatures higher than 40 °C (maximum ∼56 °C). Their habitats are often thermal mineral springs associated with volcanic systems^19^. Cyanidiales has three genera: *Cyanidium*, *Cyanidioschyzon* and *Galdieria*. The latter is composed of unicellular organisms that usually live on rocks in environments whose pH are between 0.5 and 3.0 and whose temperatures are between 50 °C and 55 °C^20^. However, Iovinella *et al*.^21^ have reported microalgae of the genus *Galdieria* in non-acid soils in Turkey. The five species of *Galdieria*, *G*. *maxima*, *G*. *partita*, *G*. *phlegrea*, *G*. *deadala* and *G*. *sulphuraria*^22,23^ stand out because of their great metabolic versatility and their abilities to grow both photoautotrophically and chemoheterotrophically in more than fifty carbon sources^24^. Heterotrophic growth favors colonization of cryptoendolithic habitats where these organisms survive in cracks of rocks with little or no light. These environments in deep areas of thermal springs and between rock fissures are extremely challenging for photosynthetic organisms because of restricted access to sunlight, but *Galdieria* thrives in these places^17,18^. Part of the reproductive success of these species is due to their tolerance of high concentrations of heavy metals and salts^25^. In addition, molecular studies have shown that horizontal transfer of genes between these organisms and archaea has facilitated their tolerance of high temperatures^26^.

The metabolic versatility of *Galdieria* combined with its capacity to grow when is pH less than 2.5 and at temperatures higher than 45 °C make the metabolites of these organisms candidates for exploitation and therefore make these organisms into important objects of study. Their metabolites include phycocyanin, a blue pigment which is thermostable at temperatures close to 73 °C and which these microorganisms can accumulate amounts between 20 and 287 times greater than those accumulated in *Spirulina platensis*^23,27,28^. Little study has been done of the proteins and polysaccharides that *Galdieria* microorganisms accumulate^29^, nor have the lipids and fatty acids that they produce been intensively investigated despite the fact that they are all of industrial interest^4^. It is known that organisms’ growth conditions influence accumulation of lipids and that production of fatty acids and lipids in microalgae is associated with stress conditions including temperature, light intensity, nitrogen source, salinity, and concentration of the carbon source^1^. In *Galdieria*, stress conditions that help generate accumulation of fatty acids can also affect the production of biomass^30^.

Given the metabolic potential of *Galdieria* and interest in obtaining PUFAs and lipids from microalgae, this study has evaluated the effects of several different culture conditions on the production of biomass, lipids and especially of PUFAs by *Galdieria* sp. USBA-GBX-832.

## Results

### Isolation and identification of USBA-GBX-832

**Figure 1:**
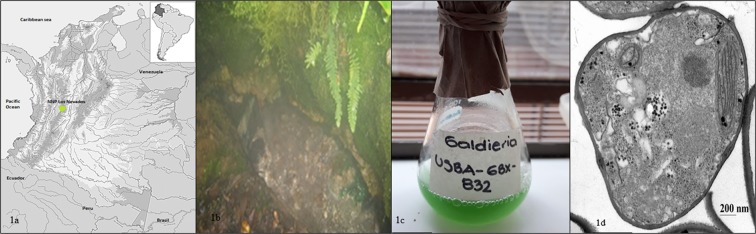
Location of the hot spring in the Los Nevados NNP and characteristics of *Galdieria* sp. USBA-GBX-832. **(a)** Location of site sampled in the Colombian Andes. (**b**) Photographs of the acidic hot spring A1. (**c)** Autotrophic culture of *Galdieria* sp. USBA-GBX-832. **(d)** Transmission electron micrograph of *Galdieria* sp. USBA-GBX-832.

USBA-GBX-832 was isolated in darkness under conditions that replicated those of thermal acidic spring A1 (Fig. 1a,b) using 250 mL of spring water enriched with oleic acid, tributyrin, tricaprylin, yeast extract and a solution of oligoelements. After 15 days of incubation at 45 °C, subcultures were created using the extinction by dilution technique in M991, a medium containing basic salts at pH 2.0. Round cells with diameters between 0.2 and 6.0 μm were obtained from the final dilution (10^−4^). Purity of the culture was corroborated through phase contrast microscopy and by observing macroscopic formation of round colonies with defined borders in the solid M991 medium at pH 3.5. During the first days of heterotrophic growth, the microalga was beige, but after 10 days of autotrophic incubation, they became green (Fig. 1c). Figure 1d shows this unicellular microalga’s architecture. USBA-GBX-832 grew in a range of pH between 0.5 and 5.0, with the optimum at 2.5. The temperature range of growth was between 20 °C and 55 °C, with the optimum at 45 °C. Photopigments activated in the presence of light turned the culture green, but in the dark it was beige. The microalga grew well in D-glucose, D-arabinose, L-arabinose and D-(+)-trehalose but grew slowly in oleic acid, tributyrin and tricaprylin.

Phylogenetic analysis based on 18S rRNA gene sequence comparison (750 bp) showed that this organism belongs to the Cyanidiales Order, with a close relation to *Galdieria sulphuraria* (92% similarity) **(**Fig. S1).

### *Galdieria* sp. USBA-GBX-832 is able to grow under autotrophic, heterotrophic, and mixotrophic conditions

**Figure 2:**
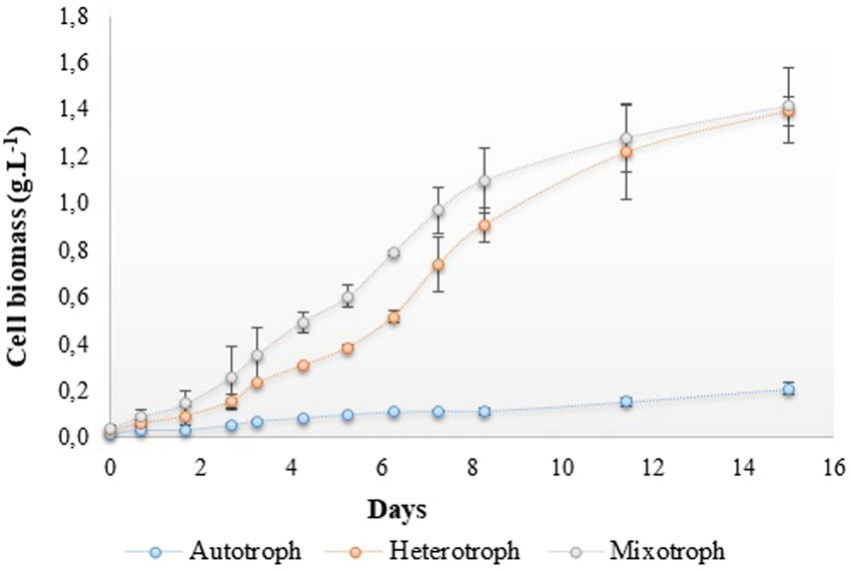
Evaluation of autotrophic, mixotrophic, and heterotrophic growth of *Galdieria* sp. USBA-GBX-832. Data are the means ± SD of three independent samples.

Growth of *Galdieria* sp. USBA-GBX-832 under heterotrophic, mixotrophic and autotrophic conditions corroborates its metabolic plasticity (Fig. 2). Use of glucose as the sole carbon source favored heterotrophic and mixotrophic growth of up to 12 times more biomass than growth under autotrophic conditions (Table 1). Heterotrophic and mixotrophic growth showed no statistically significant differences with values for μ of 0.14 and 0.16 [d^−1^], respectively. In contrast, the observed rate of growth under autotrophic conditions was ten times slower than under the other conditions (μ 0.015 [d^−1^]) (*p* 0.002). The concentration of biomass was 1.03 ± 0.06 mg.mL^−1^ from mixotrophy and 0.94 ± 0.04 mg.mL^−1^ from heterotrophy but only 0.24 ± 0.02 mg.mL^−1^ from autotrophy. The productivities of the biomass under heterotrophic and mixotrophic conditions were so similar that statistical analyses showed no significant differences (mixotrophy 68.56 ± 3.66 mg. L^−1^.d^−1^ and heterotrophy 62.88 ± 7.66 mg. L^−1^.d^−1^). In contrast, the large difference with autotrophy (15.82 ± 1.13 mg. L^−1^.d^−1^) was statistically significant. These results indicated that an organic source of carbon, glucose in this case, leads to greatest accumulation of biomass between the eighth and eleventh day of cultivation under heterotrophic and mixotrophic conditions. In contrast, this occurred on the third day under autotrophic conditions, and peak production was in any case much lower than those under the other two conditions.

**Table 1:** Evaluation of autotrophic, heterotrophic and mixotrophic growth, biomass productivity, lipid productivity and lipid content of *Galdieria* sp. USBA-GBX-832.

Condition	Carbon Source	Light	Specific growth rate µ	Biomass mg.mL^−1^	Biomass productivity (mg.L^−1^.d^−1^)	Lipid Productivity (mg.L^−1^.d^-1^)	Total lipid content (%)	Fatty acid (%)	% PUFAs*
Autotrophic	CO_2_	yes	0.015	0.24 ± 0.016	15.82 ± 1.13	2.13 ± 0.0	15.34 ± 3.3	16.66 ± 2.7	16.25 ± 0.63
Heterotrophic	Glucose 25 mM	not	0.14	0.94 ± 0.039	62.88 ± 7.66	2.29 ± 0.075	3.64 ± 0.25	36.75 ± 5.5	22.2 ± 1.84
Mixotrophic	Glucose 25 mM	yes	0.16	1.03 ± 0.06	68.56 ± 3.66	2.69 ± 0.02	3.85 ± 0.13	15 ± 2.1	23.75 ± 1.34

^*^% mg PUFAs.

Data are the mean ± SD of three independent samples.

The amount of total lipids recovered from the three culture conditions ranged between 4.0 and 5.0 mg without significant differences (*p* 0.015). However, the total lipid content was higher under autotrophy where it reached 15.3% w/w of dry biomass. Under heterotrophy and mixotrophy, it was around 3.7% w/w of dry biomass.

The composition and concentration of fatty acids obtained under autotrophic culture conditions differed significantly from those obtained from heterotrophic and mixotrophic conditions which were similar to each other (Table 2). Seventy-five percent of the fatty acids produced under autotrophic conditions were saturated, mostly palmitic acid (C_16:0_, 27.9%) and stearic acid (C_18:0_, 24.3%). Although no monounsaturated fatty acids (MUFAs) were detected, PUFAs including linoleic acid (C_18:2_, 17%) were detected.

**Table 2:** Analysis of fatty acid composition of *Galdieria* sp. USBA-GBX-832 under autotrophic, mixotrophic, and heterotrophic conditions.

Fatty acid	Mixotrophic	SD	Heterotrophic	SD	Autotrophic	SD
C14:0	0.9	0.01	1.2	0.03	—	
C15:0	0.4	0.02	0.7	0.2	11.5	0.0
C16:0	27.9	1.68	26.8	5.8	27.9	6.8
C17:0	0.8	0.02	1.1	0.36	11.1	0.0
C18:0	9.26	0.51	16.2	2.14	24.3	1.4
C20:0	0.8	0.03	0.7	0.05	0	0
**Σ Saturated**	**39.7**		**46.7**		**74.8**	
C14:1 _[cis-9]_	0.3	0.00	0.5	0.08	-	
C16:1 _[cis-9]_	0.8	0.23	0.5	0.09	0	0
C17:1 _[cis-10]_	0.3	0.00	0.6	0.16	0	0
C18:1 _[trans-9]_	0.6	0.02	1	0.19	0	0
C18:1 _[cis-9]_	34.1	3.59	28.2	2.91	0	0
C20:1 _[cis-11]_	0	1.01	0.7	0.09	0	0
**Σ MUFAs**	**36.1**		**30.7**		**0**	
C18:2 _[cis-9,12]_	22.8	1.02	20.9	2.2	16.7	0.0
C18:3 _[cis-9,12,15]_	0.8	0.02	0.5	0.09	0	
C20:2 _[cis-11,14]_	1.1	0.02	2.1	0.27	0	
**Σ PUFAs**	**24.7**		**23.5**		**16.7**	

Values are means ± SD (n = 3) (Data are given as percentage of total fatty acid).

Saturated fatty acids, especially palmitic acid (~27%), accounted for 40% to 47% of fatty acids under heterotrophic and mixotrophic conditions. The most abundant MUFA was oleic acid (C_18:1_ [cis-9]). It accounted for 28.2% under heterotrophic conditions, and 34.1% under mixotrophic conditions. Linoleic acid was the predominant PUFA C_18:2_ [cis-9, 12], accounting for ~21% in both cases. Long chain fatty acids such as eicosadienoic acid (C_20:2_ [cis-11,14]), 5,8,11-eicosatrienoic acid (C_20:3_ [cis 5,8,11]) and 15-tetracosenoic acid (C_24:1_ [cis-15]) were also found in the heterotrophic and mixotrophic cultures. The last two were detected only in trace concentrations of less than 0.25%.

### *Galdieria* sp. USBA-GBX-832 accumulates higher concentrations of PUFAs at low temperatures under heterotrophic conditions

These results show that both heterotrophic and mixotrophic conditions favor production of biomass and of MUFAs and PUFAs more than autotrophic conditions do. Given the similarity of results from heterotrophic and mixotrophic conditions, we chose to use heterotrophic conditions rather than both conditions for the following experiments.

Growth of microalga was evaluated at two temperatures, 45 °C (optimal temperature) and 25 °C (sub-optimal temperature) using nine carbon sources. The results showed that both temperature and substrate affect production of biomass, accumulation of lipids and the fatty acid profile (*p*, 0.000) (Table 3, Table S1). *Galdieria* sp. USBA-GBX-832 grew on hexoses, pentoses, disaccharides and triols. The highest concentrations of biomass were obtained with sucrose and galactose at 45 °C and glucose and galactose at 25 °C while the lowest concentrations of biomass at the two temperatures were observed with arabinose and glycerol.

**Table 3:** Biomass concentration (mg.L^−1^), biomass productivity (mg.L^−1^.d^−1^), lipid content (%), lipid productivity (mg.L^−1^.d^−1^) and PUFAs (%) of *Galdieria* sp. USBA-GBX-832 in different organic carbon sources and at different temperatures.

Temperature	Carbon source		Biomass (mg.L^−1^)		Biomass Productivity (mg.L^−1^.d^−1^)		Lipid content (%)		Lipid Productivity (mg.L^−1^.d^−1^)		PUFA (%)	
25 °C	Class	Substrate		SD		SD		SD		SD		SD
	Hexose	Glucose	1.79	0.1	89.46	4.92	5.54	0.1	4.99	0.09	35.23	0.82
		Galactose	1.8	0.16	90.17	8.01	2.6	0.2	2.34	0.27	41.63	0.90
	Hexiol	Mannitol	1.47	0.18	73.61	9.09	4.31	0.1	3.18	0.45	34.43	1.76
		Sorbitol	1.62	0.13	81.17	6.55	6.75	0.34	5.48	0.56	35.42	0.46
	Pentose	Arabinose	0.69	0.29	34.32	14.51	1.97	0.26	0.66	0.23	33.57	1.49
		Xylose	1.28	0.28	64.21	13.75	6.7	0.38	4.29	0.86	32.24	0.40
	Disaccharide	Sucrose	1.52	0.02	76.11	0.97	4.45	0.32	3.39	0.26	41.54	1.16
		Trehalose	1.63	0.11	81.6	5.64	5.97	0.15	4.88	0.45	40.68	1.06
	Triol	Glycerol	1.04	0.4	51.76	9.87	4.5	0.8	2.35	0.95	33.46	0.70
45 °C	Hexose	Glucose	1.33	0.11	165.95	14.15	2.82	0.15	4.67	0.15	25.10	0.59
		Galactose	1.61	0.00	200.65	0.15	2.61	0.11	5.23	0.23	24.76	1.20
	Hexiol	Mannitol	1.40	0.12	175.3	14.8	2.67	0.1	4.68	0.22	23.17	0.89
		Sorbitol	1.22	0.01	152.4	1.5	1.34	0.35	2.03	0.51	22.47	0.50
	Pentose	Arabinose	0.96	0.03	119.6	3.2	2.43	0.25	2.9	0.22	19.51	1.28
		Xylose	1.25	0.05	155.95	5.65	3.42	0.54	5.31	0.66	22.86	0.40
	Disaccharide	Sucrose	1.84	0.01	230.15	0.05	1.88	0.00	4.33	0.00	21.63	1.09
		Trehalose	1.08	0.05	135.5	6.7	2.92	0.45	3.94	0.41	19.71	0.58
	Triol	Glycerol	0.56	0.07	69.9	9.2	1.73	0.35	1.23	0.4	25.23	1.59

Data are the means ± SD of three independent samples.

On the third day of the stationary phase, the biomass oscillated within the narrow range of 0.69 ± 0.29 and 1.84 ± 0.01 mg.L^−1^ for all combinations of temperature and substrates. At 45 °C productivity was between 69.9 ± 9.20 and 230.15 ± 0.05 mg.L^−1^.d^−1^, while at 25 °C productivity was between 34.32 ± 14.51 and 90.17 ± 8.01 mg. L^−1^.d^−1^. Greatest productivity was obtained with galactose at 25 °C and with sucrose at 45 °C **(**Table 3**)**.

Lipid content was higher at 25 °C than it was at 45 °C. At 25 °C, it ranged between 1.97 ± 0.26% in arabinose and 6.75 ± 0.34% in sorbitol, while at 45 °C, the lipid content ranged between 1.34 ± 0.35% in sorbitol and 3.42 ± 0.54% in xylose. Productivity of lipids at both temperatures was higher in sorbitol, galactose and xylose (~5.5 mg. L^−1^.d^−1^). Glycerol produced the smallest amounts of lipids at both 25 °C and 45 °C. This may be associated with the small amount of biomass produced by this substrate.

**Figure 3:**
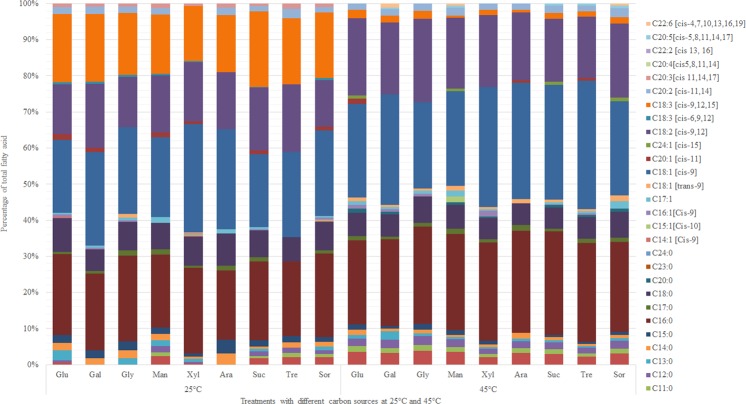
Fatty acid profiles (percentage of total fatty acids) of total lipids of *Galdieria* sp. USBA-GBX-832 under different growth conditions at 25 °C and 45 °C (Glu: Glucose; Gal: Galactose; Gly: Glycerol; Man: Mannitol; Xyl: Xylose; Ara: Arabinose; Suc: Sucrose; Tre: trehalose; Sor: Sorbitol). Data are the means ± SD of three independent samples.

Finally, at 45 °C higher concentrations of saturated fatty acids were observed. They ranged from 42.3% ± 2.45 to 48.6% ± 3.27 of total fatty acids (Fig. 3). At 25 °C the range was 31.8% ± 0.32 to 41.6% ± 1.44. Again, palmitic acid accounted for the largest portion at both temperatures (between 19.04% ± 0.29 and 27.7% ± 0.58). The concentration of MUFAs was higher at 45 °C, between 25.1% ± 3.15 and 37.4% ± 2.73, whereas at 25 °C it was 20.3% ± 0.032 to 30.7% ± 0.58. At both temperatures, the largest portion was oleic acid. In contrast, the highest concentrations of PUFAs were found at 25 °C, ranging from 32.5% ± to 41.7% ± %. At 45 °C concentrations were 20.1% ± 0.0 to 26.3% ± 0.23%. The most relevant change in the fatty acid profile was the increased concentration of α-linolenic acid (C_18: 3_ [cis-9,12,15]) at 25 °C. At 45 °C, linoleic acid (C_18: 3_ [cis-9,12]) had the highest concentration among the polyunsaturated acids.

### The production of biomass, lipids and PUFA under heterotrophic conditions changes depending on other culture variables

Other culture variables found to affect biomass production and lipid accumulation are temperature, pH, concentration of nitrogen and carbon source. Experimental design used to evaluate them are in Table S2. The ANOVA results for biomass, lipids and PUFA percentages of recovered lipids are in Table S3a–c.

Biomass productivity varied between 7 mg.L^−1^.d^−1^ and 229.8 mg.L^−1^.d^−1^. This maximum production was obtained in treatment 30 at 45 °C (Table 4) in which the concentration of galactose and its interaction with temperature had the greatest positive effects on biomass production (*p* ≤ 0.0001) **(**Fig. S2a).

**Table 4:** Effect of different culture variables on biomass and lipid productivity and fatty acid percentage of *Galdieria* sp. USBA-GBX-832 under heterotrophic conditions at 25 °C and 45 °C.

Treatment	Biomass Productivity (mg.L^−1^.d^−1^)		Lipid productivity (mg.L^−1^.d^−1^)		Σ % Saturated fatty acid. SFA		Σ % Monounsaturated fatty acid MUFA		Σ % Polyunsaturated fatty acid PUFA		Treatment	Biomass Productivity (mg.L^−1^.d^−1^)		Lipid productivity (mg.L^−1^.d^−1^)		Σ % Saturated fatty acid. SFA		Σ % Monounsaturated fatty acid MUFA		Σ % Polyunsaturated fatty acid PUFA	
	25 °C											45 °C^a^									
		SD		SD		SD		SD		SD			SD		SD		SD		SD		SD
1	16.1	1.2	1.0	0.1	35.1	0.1	26.9	0.1	38.0	0.0	17	21.3	2.5	0.8	0.3	49.6	1.1	18.3	0.5	32.1	0.6
2	16.7	2.0	1.1	0.4	36.4	0.2	25.6	0.1	37.9	0.1	18	12.4	0.3	0.8	0.1	43.3	0.3	23.7	0.7	33.0	1.0
3	59.2	1.6	2.5	0.1	34.7	0.3	29.5	0.1	35.8	0.2	19	111.2	4.9	5.2	0.2	33.2	0.2	42.8	0.3	24.0	0.1
4	82.0	14.8	5.7	0.6	33.5	0.4	32.2	2.2	34.1	2.0	20	128.0	7.1	8.3	1.8	22.3	1.6	66.1	0.6	11.6	1.0
5	40.1	1.1	1.9	0.2	38.9	0.4	32.7	0.7	28.2	0.0	21	127.8	1.6	10.1	1.5	38.8	0.5	36.4	1.2	24.1	0.4
6	46.8	6.2	1.8	0.2	38.6	0.4	33.9	1.2	27.4	0.6	22	142.9	2.1	7.3	1.1	34.1	0.4	43.6	0.9	22.3	0.5
7	19.3	2.1	1.3	0.1	28.1	0.1	43.7	0.2	27.3	1.7	23	27.7	0.4	2.0	0.2	42.6	0.7	27.5	0.9	29.9	0.2
8	7.0	1.1	0.5	0.1	39.9	2.1	33.8	0.2	25.0	4.3	24	16.7	0,0	1.0	0.2	39.6	1.4	32.4	0.9	28.1	0.5
9	37.7	2.2	2.1	0.1	32.6	0.7	26.5	0.1	40.8	0.7	25	177.3	39.6	4.8	0.2	37.0	0.7	40.8	0.6	24.0	1.1
10	13.5	1.4	1.1	0.1	42.3	0.8	34.2	1.3	23.5	0.5	26	110.3	10.6	4.5	0.6	47.9	0.5	46.9	0.5	5.2	0.9
11	11.8	0.6	0.4	0.1	35.8	0.4	26.7	0.9	37.5	0.5	27	18.2	1.1	1.6	0.4	48.0	1.2	25.1	0.8	26.9	2.0
12	17.9	1.0	0.7	0.1	50.0	0.8	47.4	0.0	3.0	0.4	28	24.5	1.1	2.5	0.2	46.6	0,0	25.3	0.7	28.2	0.7
13	20.8	0.5	1.2	0.1	37.3	0.2	32.0	0.3	30.7	0.5	29	152.3	14.4	5.7	0.6	36.0	1.2	29.1	1.6	34.9	2.8
14	31.6	1.3	1.8	0.1	31.9	0.3	29.9	0.7	38.2	0.4	30	229.8	1.0	4.5	0.1	36.7	0.8	37.2	1.2	26.1	0.3
15	16.6	2.0	0.6	0.0	34.4	0.2	30.8	0.8	34.9	0.6	31	25.2	0.9	2.4	0.3	41.1	0.3	23.8	0.0	35.1	0.2
16	11.8	1.1	0.8	0.1	42.1	2.6	6.8	2.2	51.1	0.4	32	13.9	0.3	1.4	0.3	44.0	0.1	27.9	0.5	28.1	0.3

Data are the means ± SD of three independent samples.

Total lipid productivity varied between 0.4 mg.L^−1^.d^−1^ and a peak of 10.1 mg.L^−1^.d^−1^ reached in treatment 21 at 45 °C (Table 4). The concentration of galactose repeatedly demonstrated positive effects on the model (*p* ≤ 0.000), unlike pH which had no effect on lipid productivity (*p* > 0.1266) (Fig. S2b). The PUFA percentage varied between 3.0% and 51.1% with a peak found in treatment 16 at 25 °C in which galactose concentration and nitrogen sources (yeast extract and (NH_4_)_2_SO_4_) were all at their lowest levels (Fig. S2c). This treatment created stress conditions with respect to the optimal growth conditions previously identified.

**Figure 4:**
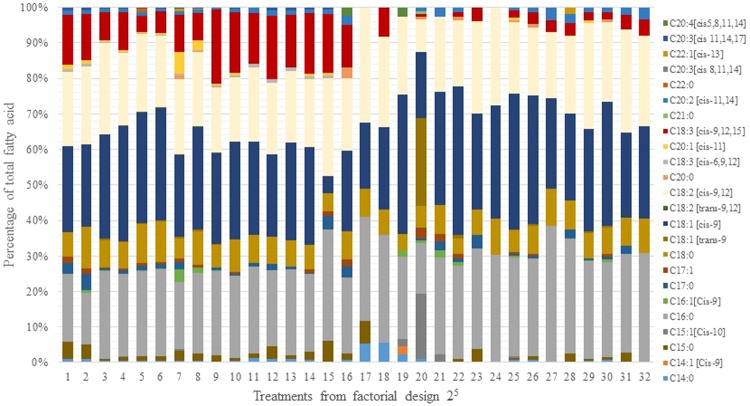
Fatty acid profile of *Galdieria* sp. USBA-GBX-832 in heterotrophic conditions. The treatments of factorial design are described in Table S2. Data are the mean ± SD of three independent samples.

Figure 4 shows specific distributions of fatty acids obtained from all treatments. No differences were observed for saturated fatty acids between 25 °C and 45 °C, but the proportions of MUFAs at 45 °C (18% to 66%) were higher than at 25 °C (6% to 47%). The opposite occurred with PUFAs which oscillated between 3% and 51% at 25 °C and between 5.2% and 35% at 45 °C. At 25 °C, the most common saturated fatty acids were palmitic acid (19–39%) and stearic acid (0.9–11.2%). Oleic acid, with concentrations between 5% and 33%, was the most common MUFA. The predominant PUFAs were linoleic acid (C_18: 2_ [cis 9,12]) (18% to 29%) and linolenic acid (C_18:3_ [cis 9,12,12]) (4.5% to 20%). The distribution of fatty acids at 45 °C was characterized by saturated acids including palmitic acid (14% to 35%) and stearic acid (4% to 10%). Oleic acid was the most common MUFA (18% to 45%) while linoleic acid was the most common PUFA (18–32%). Interestingly, we found trace concentrations of highly unsaturated fatty acids such as arachidonic (C_20:4_) and eicosapentaenoic (C_20:5_).

This study found that greater concentrations of biomass were not associated with greater lipid production indicating an inverse relationship between the amount of biomass recovered and the total amount of lipids extracted. Similarly, the evidence shows that there is an inverse relationship between the percentage of total lipids obtained and the percentage of PUFAs obtained.

Our observations show that, of all the variables evaluated, temperature had the most significant influence on the composition of fatty acids and production of lipids while the concentration of the substrate strongly influenced growth of the microalga and the amount of biomass recovered. These results are useful for designing growth media for this microalga depending on whether the desired product is biomass or lipids.

Thin-layer chromatography (TLC) detected neutral lipids, especially triacylglycerides such as tripalmitin, in all treatments (Fig. S3). This is consistent with the high concentration of palmitic acid previously identified. A more intense band in the upper part of the TLC plates may correspond to polar lipids such as phospholipids and glycerolipids which had previously been reported in *Galdieria sulphuraria*^31^.

### Effect of yeast extract on the production of lipids by *Galdieria* sp. USBA-GBX-832

Lipid extracts from cultures with yeast extract had a strong yellow coloration while cultures without the yeast extract were beige. Nevertheless, the gas chromatography (GC) fatty acid profiles showed no differences in fatty acids. For this reason, we qualitatively evaluated the lipids in *Galdieria* sp. USBA-GBX-832 that had been cultivated under optimal conditions for biomass production which was labelled treatment A (which coincides with treatment 30) with the lipids from the biomass grown in Treatment B which had no yeast extract but was otherwise identical. TLC plates were used to identify the following polar lipids in both treatments: phosphatidylethanolamine phospholipids, phosphatidylinositol, phosphatidylcholine and lysophosphatidylcholine. A comparison of treatments A and B showed that there were a greater number of bands corresponding to phospholipids in treatment B than in treatment A, but they could not be fully identified by TLC. Although the LC/MS analyses showed no differences between the two treatments, and PUFAs were observed in both, these analyses detected other fatty acids including fatty acid amide; two glycolipids, monoacylglyceride (18: 2), and digalactosyldiacylglyceride (34: 2); and sterols including ergosterol (C_28_H_44_O); and glycerophospholipids (Table 5). Three closely related compounds were found among the fatty acid amides: one with molecular formula C_18_H_35_NO that is associated with octadecenamides, C_18_H_33_NO_2_ which is associated with hydroxy octadecenamides, and C_18_H_35_NO which is associated with hexadecenamides. A porphyrinic compound with molecular formula C_35_H_36_N_4_O_5_ was also detected. Comparison of its mass and UV spectra with those reported in the Natural Products Dictionary^32^ showed that it corresponds to pheophorbide A. This pigment was reported in *Galdieria sulphuraria* during the early stationary phase of growth (∼5 d.) when this microalga was cultured under acidic conditions^33^. These results pose another challenge for the future: that of identifying the effect of the nitrogen source (yeast extract) on production of fatty acids and other metabolites of interest.

**Table 5:** Lipid profile of *Galdieria* sp. USBA-GBX-832 determined through LC-MS analyses.

Compound	Molecular Formula	Molecular weight (DB) g/mol	Mass Error (ppm)	Observed Ion	Description
***Fatty acid***					
Fatty Acid (18:3)	C_18_H_30_O_2_	278.2246	1	[M + NH_4_]^+^	Consumption of N-3 PUFA is associated with reduction of cardiovascular risk and hypercholesterolemia^57^.
Fatty Acid (18:2)	C_18_H_32_O_2_	280.2402	2,7	[M + NH_4_]^+^	
Fatty Acid (20:3)	C_20_H_34_O_2_	306.2559	5	[M + Na]^+^	
Fatty Acid (20:4)	C_20_H_32_O_2_	304.2402	2	[M + H]^+^	
Fatty Acid (20:5)	C_20_H_30_O_2_	302.2246	2	[M + H]^+^	
Fatty Acid (22:6)	C_22_H_32_O_2_	328.2402	3	[M + H]^+^	
***Fatty acid Amide***					
Hydroxyoctadecenamide (18:2)	C_18_H_33_NO_2_	295.2511	1	[M+H]^+^	Fatty acid amides are important in the structure of the ceramides and the sphingolipids. Fatty acid amides may become important for treatment of human diseases such as inflammation, pain, drug addition, eating disorders, anxiety and depression^58^.
Octadecenamide (18:1)	C_18_H_35_NO	281.2719	0,9	[M+H]^+^	
Hexadecenamide (16:1)	C_18_H_35_NO	281.2719	1	[M+H]^+^	
***Glycolipids***					
Acylglyceride (18:2)	C_21_H_38_O_4_	354.2770	1,9	[M+NH_4_]^+^	The diacylglycerides are esters of glycerol in which two of the hydroxyl groups are esterified with long chain fatty acids. They have antitumor and anti-inflammatory properties^59^.
Digalactosyldiacylglyceride DGDG (34:2)	C_49_H_88_O_15_	916.6123	1	M+NH4	
Monogalactosyldiacylglyceride MGDG (34:2)	C_43_H_78_O_10_	754,5595	1	M+Na] + / [M + NH4] +	
***Sterol***					
Sterol	C_28_H_40_O	392.3079	0,02	[M+H]^+^ [M-H2O]^+^	These compounds are involved in the synthesis of steroids. They have immunosuppressive, anti-inflammatory, antiviral and antitumor activities^60^.
Sterol	C_28_H_42_O	394.6325	4,7	[M+H]^+^ /[M + H-H2O] +	
Ergosterol	C_28_H_44_O	396.3392	0,1	[M+H]^+^ /[M-H-H2O^]+^	
Sterol	C_29_H_46_O	410.6749	1,2	[M + H]^+^	
***Glycerophospholipids***					
Diacyl-phosphatidylcoline (34:2)	C_42_H_80_NO_8_P	758.5694	1	[M + H]^+^	Phospholipids are used in pharmaceutical and cosmetics as wetting agents, emulsifiers, and builder or components of mesophases like liposomes^61^.
Diacyl-phosphatidylcoline (36:3)	C_44_H_82_NO_8_P	783.5788	1,3	[M+H]^+^	
Diacyl-phosphatidylcoline (34:1)	C_42_H_82_NO_8_P	759.5778	1	[M+H]^+^	
***Other compounds***					
Pheophorbide	C_35_H_36_N_4_O_5_	592.2686	6	[M+H]^+^	It is photodynamically toxic against mosquito larvae and fish parasite in aquatic ecosystem. Possible uses in therapies against cancer and can be considered as an antioxidant agent^62–67^^.^

## Discussion

We isolated USBA-GBX-832 strain from water and sediment samples from a hot spring in the Los Nevados NNP in Colombia and subsequently identified it as *Galdieria* sp. This was the first report of thermoacidophilic microalgae in acidic thermal springs at 3500 m above sea level. This organism has common phenotypic characteristics of the *Galderia* genus including heterotrophic, mixotrophic and autotrophic growth at acid pH and high temperatures^34^. However, similarity analysis of the 18S gene sequence suggests that this microalga could be considered a new species. Further analysis will undoubtedly be required to define whether this strain constitutes a new species within the genus *Galdieria*.

Some of the culture conditions for *Galdieria* sp. USBA-GBX-832 evaluated produced significantly larger amounts of biomass and lipids, especially PUFAs, than other conditions did. The concentrations of biomass obtained initially under mixotrophic, heterotrophic and autotrophic conditions were lower than those reported by Sakurai *et al*.^35^ for *Galdieria sulphuraria* 074W under similar culture conditions (3.8, 2.4 and 0.24 mg.mL^−1^, respectively). Nevertheless, with different treatments under heterotrophic conditions, the biomass concentration increased from 0.94 to 3.0 mg.mL^−1^. The treatments yielding increased biomass concentrations included yeast extract in the culture medium. This favored growth of the microalga. The yeast extract has a total nitrogen content that is between 10% and 12% according to its product specifications which can contribute to production of compounds such as purines, pyrimidine and B vitamins that are necessary for growth of this microalga^36^. As mentioned above, further study of the effects of this nitrogen source on the growth and accumulation of metabolites in *Galdieria* sp. USBA-GBX-832 could be important.

Interestingly, we observed that the proportion of lipids to biomass produced by USBA-GBX-832 was higher than others reported for the *Galdieria* genus. Graziani *et al*.^29^ found that *Galdieria sulphuraria* 064/309 produced 1.4% (w/w) of lipids per biomass under heterotrophic conditions and 1.1% (w/w) under autotrophic conditions whereas this study found that lipids were 3.4% (w/w) per biomass under heterotrophic conditions and 15.3% (w/w) under autotrophic conditions. These differences may be due to the metabolic capacities of each strain and/or to culture conditions. In our case, the autotrophic growth of *Galdieria* sp. USBA-GBX-832 may have generated stress conditions that led to the accumulation of lipids, as has been observed by other authors^5,37^. On the other hand, it is known that one way to storage energetic reserves in *Galderia* sp. is the synthesis of glycogen in presence of glucose, as it happens in mixotrophic and heterotrophic conditions as reported by Sakurai *et al*.^35^ and when there are no traces of sugar, the energetic reserve is the lipid synthesis, that may occur in autotrophic conditions. This may explain also, why the higher amount of lipids in the autotrophic condition. All these facts indicate that a better understanding of the metabolic dynamics of microalgae is necessary with respect to the induction of glycogen and lipid formation under different culture conditions.

Although the *Galdieria* genus grows on various carbon sources, productivity of biomass by different species varies on the same substrate. For example, *Galdieria* sp. USBA-GBX-832 produced less biomass from glycerol than did *Galdieria* *sulphuraria* 074G^38^. On the other hand, substrates such as sucrose and galactose generated greater biomass productivity by *Galdieria* sp. USBA-GBX-832. The metabolic versatility that allows growth on various carbon sources may be due to a broad spectrum of sugar kinase and polyol dehydrogenase enzymes required for introducing sugar alcohol into the heterotrophic metabolism^20^.

All of the small number of studies of lipid content produced by microalgae of the order Cyanidiales that have been published show low levels of production. For example, *Cyanidioschyzon merolae* produces ~2% (w/w) of lipids. In this case, carbon is stored mainly in the form of proteins^39^. Although some authors consider that the genus *Galdieria* only produces small amounts of lipids^4^, the results obtained in this study demonstrate the opposite. Under autotrophic conditions, total lipid of 15.3% (w/w) were reached. This is higher than any other reports for the genus or for the family. In addition, the use of galactose as a carbon source resulted in a 25 fold increase in productivity of total lipids, reaching 10.1 mg.L^-1^.d^−1^. Changes of the culture conditions and/or stress conditions also led to increasing percentages of PUFAs up to a maximum of 51.1% under heterotrophic conditions at 25 °C. Polyunsaturation of fatty acids often occurs in cultures at low temperatures because it gives greater fluidity to the cell membrane^40^. This could explain the increase in linolenic acid concentration (C_18:3_) at 25 °C.

The fatty acid profile of the genus *Galdieria* varies according to species and culture conditions as reported by various authors^41^. In *Galdieria* sp. USBA-GBX-832 palmitic acid (C_16:0_), oleic acid (C_18:1_) and linoleic acid (C_18:2_) predominated at 45 °C, while at 25 °C the same acids plus linolenic acid (C_18:3_) were predominant. Oleic acid (C_18:1_) was the predominant product when glycerol was used as the carbon source for *G*. *sulphuraria* 064/309 under heterotrophic conditions, but under autotrophic conditions *G*. *sulphuraria* 064/309 mainly produced linoleic acid (C_18:2_)^29^. As mentioned, when glycerol was the carbon source, *Galdieria* sp. USBA-GBX-832 produced the smallest amount of lipids. Vítová *et al*.^31^ found that *G*. *sulphuraria* 002 (Galdieri) mostly produced palmitic acid (C_16:0_) followed by linoleic acid (C_18:2_) at pH between 1.0 and 4.0. Under all culture conditions, *G*. *sulphuraria* 074W accumulated mainly palmitic acid (C_16:0_), oleic acid (C_18:1_), and linoleic acid (C_18:2_). Under mixotrophic conditions it also produced stearic acid (C_18:0_) while it also produced linolenic acid (C_18:3_) under heterotrophic conditions^35^. That study also observed trace concentrations of several long-chain PUFAs including eicosadienoic acid (C_20:2_) and dihomo-γ-linolenic acid (C_20:3_) as well as traces of docosadienoic acid (C_22:2_) while arachidonic acid (C_20:4_) and eicosapentaenoic acid (C_20:5_) were absent. In our study, trace concentrations of long-chain PUFAs such as eicosadienoic acid (C_20:2_), dihomo-γ-linolenic acid (C_20:3_) and docosadienoic acid (C_22:2_) were detected, as well as arachidonic acid (C_20:4_) and eicosapentaenoic acid (C_20:5_). One very important finding was detection of these last two fatty acids with high degrees of unsaturation, something which had not previously been reported for the genus despite the abundance of PUFAs within the phylum Rhodophyta (with the exception of Cyanidales)^42^. As mentioned before, synthesis of PUFAs can occur along two pathways, an aerobic pathway and an anaerobic pathway. The former involves desaturase and elongase enzymes while in the anaerobic, polyketide synthase (PKS) allows for *de novo* synthesis of PUFAs. Desaturase enzymes were not detected in the genome analysis of *G*. *sulphuraria*^26^. Our ongoing analysis of the genome of *Galdieria* sp. USBA-GBX-832 has already demonstrated the presence of elongase enzymes but has not yet identified desaturase enzymes that are important for this PUFAs biosynthesis pathway. On the other hand, a cluster of biosynthetic genes for polyketide synthase (PKS) has been identified, and this could indicate that USBA-GBX-832 also uses the alternative route for PUFA synthesis. (Data not shown). The exact pathway or pathways of PUFA biosynthesis in *Galdieria* sp. USBA-GBX-832 remains to be determined, as does the question of whether or not these highly unsaturated fatty acids are present in other species of the genus *Galdieria*.

Finally, it is noteworthy that LC/MS analyses have made it possible to detect fatty acid amides, glycolipids and digalactosyldiacylglycerides, some of which could not be identified by comparison of their MS and UV spectra with those reported in the Dictionary of Natural Products. This opens up the possibility that *Galdieria* sp. USBA-GBX-832 accumulates previously unidentified compounds. Without a doubt, these promising results will allow us and others to continue exploring accumulation of fatty acids and other compounds of interest produced by this microalga. On the other hand, our results differ with those of the study of *Galdieria sulphuraria* 002 by Vítová *et al*.^31^ which stated that at pH of 1.0 and 2.0 the concentration of phospholipids decreased considerably while the concentrations of lipids related to betaine such as 1,2-Dipalmitoyldiacylglyceryltrimethylhomoserine (DGTS) and diacylglyceryl hydroxymethyltrimethyl-β-alanine (DGTA) compounds increased. In *Galdieria* sp. USBA-GBX-832, were not detected at pH 2.5.

Although the taxonomic status of USBA-GBX-832 as a new species of *Galdieria* will be the subject of future studies, we already know that *Galdieria* sp. USBA-GBX-832 presents special characteristics such as the accumulation of PUFAs that had not previously been reported for this genus and that differentiate it from other species of the genus. This study has demonstrated key relationships between growth conditions, biomass production, lipid production and fatty acid composition. Growth conditions that led to greater accumulation of biomass, lipids and PUFAs were also identified. Temperature had an important effect on accumulation of PUFAs, the largest proportion of which consisted of linoleic acid and α-linolenic acid (~20%). Similarly, this study has shown that, under heterotrophic conditions at lower temperatures, there is less accumulation of biomass, but greater production of PUFAs. These findings are similar to those of Chaisutyakorn *et al*.^43^ In the future, we propose to evaluate increases in biomass production using the multiple-stage process technique evaluated by Wan *et al*.^27^ This could lead to sequential increases in biomass production under the previously identified conditions. The culture would then be submitted to stress conditions for increased production of lipids and PUFAs. Our ongoing analysis of the genome of USBA-GBX-832 may confirm the presence of pathways for synthesis of polyunsaturated acids identified in *Galdieria* sp. USBA-GBX-832. Finally, this study is just the beginning of what promises to be a number of very fruitful studies of *Galdieria* sp. USBA-GBX-832 as a source of bioactive compounds.

## Methods

### Isolation and identification of USBA-GBX-832

We isolated a thermoacidophilic microalga from sediment and water samples collected from an acidic hot spring (A1) located in Los Nevados National Natural Park (NNP) (04 ° 58'13.2“N, 75 ° 22'42“W) in Colombia at ~3500 m above sea level. We stored it in sterile glass containers and subsequently named this strain USBA-GBX-832. The temperature at the sampling point was 57 °C, and the pH was 2.3. Samples were transported to the laboratory where they were treated immediately. The growth medium was basal 991 medium^44^ supplemented with 5 mM oleic acid, 0.1% (v/v) tributyrin and tricaprylin, 0.05 g.L^−1^ yeast extract and 1 mL.L^−1^ of oligoelements solution^45^. Culturing conditions were 45 °C and pH 2.0 ± 0.2. Pure culture was obtained by the extinction dilution technique^46^.

We identified USBA-GBX-832 through analysis of the 18S rRNA gene sequence. DNA was extracted with the Wizard® Genomic DNA kit (Promega cat # A1120) according to the manufacturer’s instructions from 50 mL of an exponentially growing microbial culture at 8,000 *g* for 10 minutes. Two primers were used: 18SR (5’-GTCAGAGGTGAAATTCTTGGATTTA-3’) and 18SF (5’-AGGGCAGGGACGTAATCAACG-3’). All PCR reactions contained 20 to 50 ng of DNA template, 0.2 μM of each primer, 0.1 mM dNTPs, 1.5 mM MgCl_2_, 1X Buffer and 0.5 U of GoTaq® DNA Polymerase in a 50-µL total volume reaction (Promega). PCR conditions were established according to the amplification protocols of Toplin *et al*.^47^. Sequencing was performed using an ABI PRISM® 3500 (DNA sequencing laboratory, Universidad de Los Andes, Bogotá, Colombia). Raw sequence data were imported into BioEdit, version 7.2.5, sequence editor^48^ and corrected manually for errors. Sequences were compared against sequences of type strains using BLAST from NCBI (http://www.ncbi.nlm.nih.gov/)^49^. MUSCLE (Multiple Sequence Comparison by Log- Expectation)^50^ was used to compare this sequence with other 18S rRNA sequences from *Galdieria* strains and MEGA 7.0.25 (Molecular Evolutionary Genetics Analysis) software was used for maximum likelihood phylogenetic tree reconstruction. Bootstrap values were based on 1,000 resamplings^51^.

Cellular morphology was determined by electron microscopy as described by Watson *et al*.^52^. The strain USBA-GBX 832 was deposited in the *Colección de Microorganismos de la Pontificia Universidad Javeriana* (WDCM 857) under identification number CMPUJ U832.

### Nucleotide sequence accession number

The 18S rRNA gene sequences of strain USBA-GBX 832 were deposited in GenBank under accession number MK002974.

### Cultivation of USBA-GBX-832

USBA-GBX-832 was routinely cultured from a stock culture stored at −80 °C in the microorganism collection. Heterotrophic culturing was carried out in 250 mL Erlenmeyer flasks with 125 mL of M991 medium supplemented with 25 mM of D-glucose and 0.5 g.L^−1^ of yeast extract^44^. Cultures were incubated in the dark at 45 °C with constant stirring at 180 rpm for 5 days. Once the exponential phase was reached, it was subcultured in M991 medium at pH 2.5 without yeast extract under autotrophic conditions for a period of 8 days at 45 °C with continuous stirring at 180 rpm. The culture thus obtained was used as inoculum for experimental batch tests that will be described later. Experimental trials were initiated with cultures with optical densities (OD) between 0.020 and 0.030 at 750 nm. To determine optimal culture conditions for growth under heterotrophic conditions, we varied the temperature from 20 °C to 60 °C and the pH from 1.0 to 8.0 while monitoring the increase of biomass by measuring OD at 750 nm. All experimental tests were done in triplicate.

### Experimental tests

#### Growth of USBA-GBX-832 under conditions of autotrophy, heterotrophy, and mixotrophy

USBA-GBX-832 was incubated with continuous light at 35 μE/m^2^s for autotrophic and mixotrophic growth while heterotrophic growth occurred in darkness. To evaluate heterotrophic and mixotrophic growth, cultures were supplemented with 25 mM D-glucose. All cultures were incubated at pH 2.5 at 45 °C with continuous stirring at 180 rpm for 15 days.

#### Determination of effects of incubation temperature on heterotrophic growth of USBA-GBX-832 in various substrates

Production of biomass and accumulation of lipids and fatty acids were evaluated at two temperatures, 25 °C and 45 °C, under heterotrophic conditions using D-glucose, D-galactose, D- and L-arabinose, D-xylose, D-mannitol, D-sorbitol, D-sucrose and D - (+) - trehalose at final concentrations of 25 Mm and glycerol at a concentration of 10 g.L^−1^.

#### Factorial analysis of additional variables for growth of USBA-GBX-832

In order to identify the effect of other culture variables on biomass production and lipid accumulation we used a complete factorial design at two levels with Design expert® 9.0 software (Stat-Ease, Inc. USA). Culture variables were temperature (25 °C and 45 °C), pH (1.0 and 2.5), galactose concentration (2.5 and 25 mM), yeast extract (0 and 0.5 gL^−1^) and ammonium sulfate (0.13 and 1.3 gL^−1^). Each experimental round was conducted in triplicate **(**Table S2).

#### Evaluation of the effect of added yeast extract on production of lipids by USBA-GBX-832

Treatments A and B tested the effects of yeast in the culture medium. In treatment A, USBA-GBX-832 was cultivated under heterotrophic conditions with 0.5 g.L^−1^ of yeast extract, 1.3 g.L^−1^ of sodium chloride and 25 mM galactose. In treatment B, the same culture medium, but without yeast extract, was used. The cultures were incubated in the dark at 45 °C and at pH 2.5. They were stirred at 180 rpm, and the test was performed in duplicate.

## Analytical Methods

### Determination of biomass production and analysis of lipids

OD was measured spectrophotometrically (HACH Loveland, CO, U.S.A) at 750 nm. Absorbance values were transformed to g.L^−1^ by means of a calibration curve based on the relationship between OD and cell dry weight. Each experiment ended three days after the stationary phase began. Biomass was centrifugally concentrated at 6,000 *g* for 10 min. Subsequently, biomass was lyophilized for 24 hours in a freeze dryer (LABCONCO, FreeZone 4.5), then lipids were extracted according to a modified version of the method of Bligh & Dyer (1959)^53^.

Freeze dried cells were collected in amber glass bottles and chloroform was added for extraction. After 5 min, the solution was mixed with methanol at a ratio of 1:2 (v/v, chloroform: methanol). Cell lysis was carried out by ultrasound at an amplitude of 50% for periods of 20:10 (20 s of work and 10 s of rest) using a Qsonic ultrasonicator (Ref Q125) at a frequency of 20 kHz. Then chloroform was added until it reached a 2:2 ratio (v/v) with methanol. Subsequently, a phosphate buffer solution (0.05 M, pH 7) was added. Samples were shaken vigorously and centrifuged to separate the lipid fraction. Uninoculated culture medium was processed as a negative control. The lipids were quantified gravimetrically and stored in amber colored tubes at −20 °C. Productivity of biomass and lipids were calculated in mg.L^−1^.d^−1^.

### Determination of fatty acid profiles

Fatty acid profiles were obtained by GC (GC-2014, Shimadzu) in the form of fatty acid methyl esters (FAME). The lipids and fatty acids were derivatized with BF_3_/MeOH following the AOAC 925.41 protocol^54^. FAME were separated and quantified using a Rt-2560 column (100 m, 0.25 mmID, 0.2 μ) (Restek) with an oven temperature of 140 °C for 5 minutes. Temperature was then raised at 4 °C/min until it reached 240 °C. The carrier gas was helium. The standard FAME Mix-37 (47885U-Supelco) was used for identification of FAME by comparison of mass spectra (IE, 70 eV) obtained with an Agilent 5975B series Gas Chromatograph/Mass Spectrometer with those reported in the libraries of the National Institute of Standards and Technology (NIST) Standard Reference Database and the eighth edition of the Wiley Registry of Mass Spectral Data.

### Lipid separation by TLC

The lipids extracted were separated by TLC using various solvents. Hexane/ethyl acetate (7:3) was used for neutral lipid separation, chloroform/methanol/ammonium hydroxide (65: 25:4, v/v/v) was used for phospholipids, and toluene/methanol (7:3, v/v) was used for sphingolipids and ceramides. Two-dimensional TLC was used for glycolipids and phospholipids with chloroform/methanol/ammonium hydroxide (65:35:4, v/v/v) in the first dimension and chloroform/methanol/AcOH/water (80:9:12:2 v/v/v/v) in the second dimension. Band profiles were identified using triacylglyceride (triolein, tripalmitin, tricaprylin and tributyrin) and phospholipid (L-α-Lisophosphatidylcholine, L-α-phosphatidylcholine, L-α-phosphatidylethanolamine, L-α-phosphatidylinositol)^55,56^. The reagent for visualization used was *p*-anisaldehyde/H_2_SO_4_.

### Lipid profiling by LC-MS analysis

Samples from treatments A and B were analyzed by liquid chromatography-mass spectrometry (LC/MS) as described by González-Menéndez *et al*.^57^. The LC analysis was performed on an Agilent 1200 Rapid Resolution HPLC connected to a Bruker maXis mass spectrometer. The volume of sample injected was 2 μL. The Zorbax SB-C8 column (2.1 × 30 mm, 3.5 μm particle size) used for separation was maintained at 40 °C with a flow rate of 300 μL/min. A gradient of solvent A (10% acetonitrile and 90% water) and solvent B (90% acetonitrile and 10% water), both with 13 mM ammonium formate and 0.01% (v/v) trifluoroacetic acid, was employed as the mobile phase. The gradient started at 10% B and rose to 100% B in 6 minutes. Full diode array UV scans from 100 to 900 nm were collected in 4-nm steps at 0.25 sec/scan. Ionization was achieved by Electrospray Ionization (ESI) in positive mode. The instrumental parameters were 4 kV capillary voltage with drying gas flow of 11 L/min at 200 °C and nebulizer pressure at 2.8 bars. TFA-Na cluster ions were used for mass calibration of the instrument prior to each sample injection. Each sample was recalibrated by infusion with the same TFA-Na calibrant. The UV signal, retention time, mass signal and molecular formula of the selected ions were compared to the UV-HPLC-HRMS data of known metabolites stored in the MEDINA Foundation database and matching molecular formulas in the Chapman and Hall Dictionary of Natural Products database^32^.

Other lipid analyses were performed using an HPLC system 1200 series coupled to a 6520 QTOF mass spectrometer (Agilent Technologies, Santa Clara, CA USA). We injected 10 μL of the lipid extract into a C8 column (Phenomenex-Luna C8 150 mm × 2.0 mm, 3um), and chromatographic analysis was carried out at 60 °C using a gradient elution and application of 10 mM ammonium formate in Milli-Q water (A) and 10 mM ammonium formate in methanol (B) at a constant flow of 0.5 mL/min as previously described by Whiley *et al*.^58^. The eluent gradient started at 75% and increased to 96% B in 23 min, was held for 22 min at 96% B, and was then increased to 100% B in 1 min and kept constant for 10 min. Finally, the gradient returned to initial conditions in 1 min and was kept constant for 10 min until the system reequilibrated. Mass spectrometric detection was performed in positive ESI mode with full scan applied to a mass range from 100 to 1200 m/z. The mass spectrometer source conditions consisted of a capillary voltage of 3.5 kV, a nebulizer gas flow rate of 13.0 L/min, a source temperature of 350 °C, and a source pressure of 40 psig. During all analyses, two reference masses were continuously injected for mass correction: m/z 121.0509 (C_5_H_4_N_4_) and m/z 922.0098 (C_18_H_18_O_6_N_3_P_3_F_24_). Features were putatively identified with the CEU Mass Mediator tool (http://ceumass.eps.uspceu.es/) by matching the observed accurate mass of each compound with the m/z values available online at METLIN (http://metlin.scripps.edu), KEGG (http://genome.jp/kegg), and lipid MAPS (http://lipidMAPS.org)^59^.

### Statistical analysis

Statistical analyses included normality testing of the response variables of biomass productivity and total lipids and parametric tests of mean differences (Analysis of variance - ANOVA). The latter included the Kruskal-Wallis H-test and Post-Hoc tests of the hierarchy of differences. The statistical significance of the regression coefficients was corroborated using Student’s t-test. Surface and contour analysis was done with the R statistical package^60^ and Design expert® 9.0.

## Supplementary information

Supplementary Material
