# Supplementary material for: Production of Polyunsaturated Fatty Acids and Lipids from Autotrophic, Mixotrophic and Heterotrophic cultivation of Galdieria sp. strain USBA-GBX-832
Source: Sci Rep. 2019 Jul 25;9:10791. doi: 10.1038/s41598-019-46645-3 (PMC6658668; doi:10.1038/s41598-019-46645-3)
Supplement: Supplementary file 1 — Supplementary Material [file 41598_2019_46645_MOESM1_ESM.pdf]

**SUPPLEMENTARY MATERIAL**

# Production of Polyunsaturated Fatty Acids and Lipids from Autotrophic, Mixotrophic and Heterotrophic cultivation of *Galdieria* sp. strain USBA-GBX-832

López Gina[1], Yate Camilo[2], Ramos Freddy A.[3], Cala Mónica P. [4], Restrepo Silvia[5], Baena Sandra[1]

[1] Unidad de Saneamiento y Biotecnología Ambiental (USBA), Departamento de Biología, Pontificia Universidad Javeriana, POB 56710, Bogotá DC, Colombia.

[2]Facultad de estadística, Universidad Santo Tomas, Carrera 9 # 51-11, Bogotá DC, Colombia.

[3]Departamento de Química, Universidad Nacional de Colombia-Sede Bogotá, Carrera 30 # 45-03, Bogotá DC, Colombia.

[4]Department of Chemistry, Universidad de los Andes, Cra 1 No. 18A-12, Bogotá DC, Colombia.

[5]Biological Sciences Department, Universidad de los Andes, Cra 1 No. 18A-12, Bogotá DC, Colombia.

***Corresponding author:*** Sandra Baena. Pontificia Universidad Javeriana, POB 56710, Bogotá DC, Colombia. Tel: + 57-1-320 83 20. Ext 4170; E-mail: baena@javeriana.edu.co

***Present/permanent address:*** Unidad de Saneamiento y Biotecnología Ambiental, Departamento de Biología, Pontificia Universidad Javeriana, POB 56710, Bogotá DC, Colombia.

**Table S1.** Analysis of variance (ANOVA) to compare the mean values obtained when *Galdieria* sp. USBA-GBX-832 was growing at different temperatures under heterotrophic conditions. SS: Sum of squares, df: Degrees of freedom; *Significance at $p < 0.05$.

| Source | Dependent variable | Sum of square | df | Mean square | F | *P* value |
|---|---|---|---|---|---|---|
| **Model** | **Biomass Productivity** | 855505,228[a] | 18 | 47528,068 | 523,493 | ,000 |
| | **Lipid productivity** | 832,804[b] | 18 | 46,267 | 221,491 | ,000 |
| | **%Lipids** | 825,174[c] | 18 | 45,843 | 419,791 | ,000 |
| | **%∑ PUFA** | 50246,217[d] | 18 | 2791,456 | 3336,328 | ,000 |
| **Temperature** | **Biomass Productivity** | 97024,080 | 1 | 97024,080 | 1068,662 | ,000 |
| | **Lipid productivity** | 1,270 | 1 | 1,270 | 6,080 | ,019 |
| | **%Lipids** | 68,394 | 1 | 68,394 | 626,293 | ,000 |
| | **%∑ PUFA** | 2568,181 | 1 | 2568,181 | 3069,471 | ,000 |
| **Substrate** | **Biomass Productivity** | 42398,940 | 8 | 5299,867 | 58,375 | ,000 |
| | **Lipid productivity** | 62,295 | 8 | 7,787 | 37,278 | ,000 |
| | **%Lipids** | 40,010 | 8 | 5,001 | 45,797 | ,000 |
| | **%∑ PUFA** | 189,491 | 8 | 23,686 | 28,310 | ,000 |
| **Temperature * Substrate** | **Biomass Productivity** | 17157,100 | 8 | 2144,637 | 23,622 | ,000 |
| | **Lipid productivity** | 46,217 | 8 | 5,777 | 27,656 | ,000 |
| | **%Lipids** | 36,495 | 8 | 4,562 | 41,774 | ,000 |
| | **%∑ PUFA** | 268,212 | 8 | 33,526 | 40,071 | ,000 |
| **Error** | **Biomass Productivity** | 3268,448 | 36 | 90,790 | | |
| | **Lipid productivity** | 7,520 | 36 | ,209 | | |
| | **%Lipids** | 3,931 | 36 | ,109 | | |
| | **%∑ PUFA** | 30,121 | 36 | ,837 | | |
| **Total** | **Biomass Productivity** | 858773,676 | 54 | | | |
| | **Lipid productivity** | 840,324 | 54 | | | |
| | **%Lipids** | 829,105 | 54 | | | |
| | **%∑ PUFA** | 50276,337 | 54 | | | |

**a. $R^2$ = ,996 (Adj R-Squared = ,994)**

**b. $R^2$ = ,991 (Adj R-Squared = ,987)**

**c. $R^2$ = ,995 (Adj R-Squared = ,993)**

**d. $R^2$ = ,999 (Adj R-Squared = ,999)**

**Table S2**. Experimental design used to evaluate other culture variables that favored higher levels of biomass production and lipid accumulation

| Test | pH | Galactose (mM) | Yeast extract (g.L$^{-1}$) | $(NH_4)_2SO_4$ (g.L$^{-1}$) | Temperature (°C) |
|---|---|---|---|---|---|
| 1 | 2.5 | 2.5 | 0.5 | 0.13 | 25 |
| 2 | 2.5 | 2.5 | 0 | 1.3 | 25 |
| 3 | 2.5 | 25 | 0 | 0.13 | 25 |
| 4 | 2.5 | 25 | 0.5 | 1.3 | 25 |
| 5 | 1.5 | 25 | 0 | 1.3 | 25 |
| 6 | 1.5 | 25 | 0.5 | 0.13 | 25 |
| 7 | 1.5 | 2.5 | 0.5 | 1.3 | 25 |
| 8 | 1.5 | 2.5 | 0 | 0.13 | 25 |
| 9 | 1.5 | 25 | 0.5 | 1.3 | 25 |
| 10 | 1.5 | 25 | 0 | 0.13 | 25 |
| 11 | 1.5 | 2.5 | 0 | 1.3 | 25 |
| 12 | 1.5 | 2.5 | 0.5 | 0.13 | 25 |
| 13 | 2.5 | 25 | 0 | 1.3 | 25 |
| 14 | 2.5 | 25 | 0.5 | 0.13 | 25 |
| 15 | 2.5 | 2.5 | 0.5 | 1.3 | 25 |
| 16 | 2.5 | 2.5 | 0 | 0.13 | 25 |
| 17 | 2.5 | 2.5 | 0.5 | 0.13 | 45 |
| 18 | 2.5 | 2.5 | 0 | 1.3 | 45 |
| 19 | 2.5 | 25 | 0 | 0.13 | 45 |
| 20 | 2.5 | 25 | 0.5 | 1.3 | 45 |
| 21 | 1.5 | 25 | 0 | 1.3 | 45 |
| 22 | 1.5 | 25 | 0.5 | 0.13 | 45 |
| 23 | 1.5 | 2.5 | 0.5 | 1.3 | 45 |
| 24 | 1.5 | 2.5 | 0 | 0.13 | 45 |
| 25 | 1.5 | 25 | 0.5 | 1.3 | 45 |
| 26 | 1.5 | 25 | 0 | 0.13 | 45 |
| 27 | 1.5 | 2.5 | 0 | 1.3 | 45 |
| 28 | 1.5 | 2.5 | 0.5 | 0.13 | 45 |
| 29 | 2.5 | 25 | 0 | 1.3 | 45 |
| 30 | 2.5 | 25 | 0.5 | 0.13 | 45 |
| 31 | 2.5 | 2.5 | 0.5 | 1.3 | 45 |
| 32 | 2.5 | 2.5 | 0 | 0.13 | 45 |

**Table S3a.** Analysis of variance (ANOVA) of the multifactorial experimental design over **biomass productivity** of *Galdieria* sp. USBA-GBX-832

| | | | | | | | |
|---|---|---|---|---|---|---|---|
| **Response** | 1 | **Biomass productivity** | | | | | |
| **Transform:** | **Natural Log** | **Constant:** | 0 | | | | |
| | | | **Analysis of variance table [Partial sum of squares - Type III]** | | | | |
| **Source** | **Sum of Squares** | **df** | **Mean Square** | **F Value** | **p-value Prob > F** | | |
| **Model** | 59.10 | 31 | 1.91 | 576.10 | < 0.0001 | **SIGNIFICANT** | |
| **A-Temperature** | 10.88 | 1 | 10.88 | 3288.17 | < 0.0001 | | |
| **B-pH** | 0.12 | 1 | 0.12 | 35.09 | < 0.0001 | | |
| **C-[Gal]** | 23.83 | 1 | 23.83 | 7201.78 | < 0.0001 | | |
| **D-Y.E** | 2.93 | 1 | 2.93 | 886.23 | < 0.0001 | | |
| **E-$(NH_4)_2SO_4$** | 0.32 | 1 | 0.32 | 95.79 | < 0.0001 | | |
| **AB** | 0.36 | 1 | 0.36 | 109.04 | < 0.0001 | | |
| **AC** | 1.19 | 1 | 1.19 | 358.59 | < 0.0001 | | |
| **AD** | 0.02 | 1 | 0.02 | 7.26 | 0.0111 | | |
| **AE** | 0.11 | 1 | 0.11 | 33.76 | < 0.0001 | | |
| **BC** | 0.43 | 1 | 0.43 | 128.44 | < 0.0001 | | |
| **BD** | 0.12 | 1 | 0.12 | 37.68 | < 0.0001 | | |
| **BE** | 0.22 | 1 | 0.22 | 65.07 | < 0.0001 | | |
| **CD** | 0.85 | 1 | 0.85 | 257.88 | < 0.0001 | | |
| **CE** | 0.87 | 1 | 0.87 | 264.41 | < 0.0001 | | |
| **DE** | 0.01 | 1 | 0.01 | 3.95 | 0.0555 | | |
| **ABC** | 0.77 | 1 | 0.77 | 232.44 | < 0.0001 | | |
| **ABD** | 0.22 | 1 | 0.22 | 67.59 | < 0.0001 | | |
| **ABE** | 0.00 | 1 | 0.00 | 1.01 | 0.3225 | | |
| **ACD** | 0.62 | 1 | 0.62 | 186.68 | < 0.0001 | | |
| **ACE** | 1.20 | 1 | 1.20 | 362.70 | < 0.0001 | | |
| **ADE** | 0.01 | 1 | 0.01 | 4.24 | 0.0477 | | |
| **BCD** | 1.32 | 1 | 1.32 | 399.48 | < 0.0001 | | |
| **BCE** | 0.59 | 1 | 0.59 | 178.20 | < 0.0001 | | |
| **BDE** | 0.45 | 1 | 0.45 | 135.28 | < 0.0001 | | |
| **CDE** | 0.92 | 1 | 0.92 | 277.55 | < 0.0001 | | |
| **ABCD** | 0.53 | 1 | 0.53 | 160.94 | < 0.0001 | | |
| **ABCE** | 1.10 | 1 | 1.10 | 332.68 | < 0.0001 | | |
| **ABDE** | 1.14 | 1 | 1.14 | 345.45 | < 0.0001 | | |
| **ACDE** | 2.82 | 1 | 2.82 | 853.23 | < 0.0001 | | |
| **BCDE** | 0.30 | 1 | 0.30 | 90.06 | < 0.0001 | | |
| **ABCDE** | 4.83 | 1 | 4.83 | 1458.36 | < 0.0001 | | |

| | | | |
|---|---|---|---|
| **Std. Dev.** | 0.057 | **R-Squared** | 0.99 |
| **Mean** | 3.55 | **Adj R-Squared** | 0.99 |
| **C.V. %** | 1.62 | **Pred R-Squared** | 0.99 |

**Table S3b.** Analysis of variance (ANOVA) of the multifactorial experimental design used to evaluate various culture factors over **lipid productivity** of *Galdieria* sp. USBA-GBX-832

| | | | | | | |
|---|---|---|---|---|---|---|
| **Response** | | **1** | **Lipid productivity** | | | |
| **Transform:** | **Natural Log** | **Constant:** | **0** | | | |
| **Analysis of variance table [Partial sum of squares - Type III]** | | | | | | |
| **Source** | **Sum of Squares** | **df** | **Mean Square** | **F Value** | **p-value Prob > F** | |
| **Model** | 54.87 | 31 | 1.77 | 71.24 | < 0.0001 | **SIGNIFICANT** |
| **A-Temperature** | 13.82 | 1 | 13.82 | 556.11 | < 0.0001 | |
| **B-pH** | 0.01 | 1 | 0.01 | 0.47 | 0.4981 | |
| **C-[Gal]** | 30.11 | 1 | 30.11 | 1211.80 | < 0.0001 | |
| **D-Y.E** | 1.67 | 1 | 1.67 | 67.07 | < 0.0001 | |
| **E-$(NH_4)_2SO_4$** | 0.52 | 1 | 0.52 | 21.05 | < 0.0001 | |
| **AB** | 0.40 | 1 | 0.40 | 16.28 | 0.0003 | |
| **AC** | 4.46 | 1 | 4.46 | 179.38 | < 0.0001 | |
| **AD** | 0.00 | 1 | 0.00 | 0.00 | 0.9729 | |
| **AE** | 0.06 | 1 | 0.06 | 2.42 | 0.1294 | |
| **BC** | 0.07 | 1 | 0.07 | 2.69 | 0.1105 | |
| **BD** | 0.19 | 1 | 0.19 | 7.50 | 0.0100 | |
| **BE** | 0.23 | 1 | 0.23 | 9.39 | 0.0044 | |
| **CD** | 0.03 | 1 | 0.03 | 1.09 | 0.3053 | |
| **CE** | 0.06 | 1 | 0.06 | 2.26 | 0.1426 | |
| **DE** | 0.00 | 1 | 0.00 | 0.11 | 0.7410 | |
| **ABC** | 0.25 | 1 | 0.25 | 10.00 | 0.0034 | |
| **ABD** | 0.11 | 1 | 0.11 | 4.23 | 0.0479 | |
| **ABE** | 0.08 | 1 | 0.08 | 3.06 | 0.0899 | |
| **ACD** | 0.09 | 1 | 0.09 | 3.75 | 0.0618 | |
| **ACE** | 0.01 | 1 | 0.01 | 0.22 | 0.6389 | |
| **ADE** | 0.03 | 1 | 0.03 | 1.12 | 0.2982 | |
| **BCD** | 0.65 | 1 | 0.65 | 26.14 | < 0.0001 | |
| **BCE** | 0.04 | 1 | 0.04 | 1.57 | 0.2199 | |
| **BDE** | 0.02 | 1 | 0.02 | 0.95 | 0.3368 | |
| **CDE** | 0.20 | 1 | 0.20 | 8.07 | 0.0078 | |
| **ABCD** | 0.07 | 1 | 0.07 | 2.84 | 0.1019 | |
| **ABCE** | 0.07 | 1 | 0.07 | 2.80 | 0.1041 | |
| **ABDE** | 0.05 | 1 | 0.05 | 1.99 | 0.1681 | |
| **ACDE** | 0.51 | 1 | 0.51 | 20.46 | < 0.0001 | |
| **BCDE** | 0.25 | 1 | 0.25 | 10.25 | 0.0031 | |
| **ABCDE** | 0.83 | 1 | 0.83 | 33.36 | < 0.0001 | |

| | | | |
|---|---|---|---|
| **Std. Dev.** | 0.16 | **R-Squared** | 0.99 |
| **Mean** | 0.61 | **Adj R-Squared** | 0.97 |

| | | | |
|---|---|---|---|
| **C.V. %** | 25.77 | **Pred R-Squared** | 0.94 |

**Table S3c.** Analysis of variance (ANOVA) of multifactorial experimental design used to evaluate various culture factors over **% PUFA** of *Galdieria* sp. USBA-GBX-832

| | | | | | | |
|---|---|---|---|---|---|---|
| **Response** | | 3 | **%PUFA** | | | |
| **Transform:** | Natural Log | | Constant: | 0 | | |

**Analysis of variance table [Partial sum of squares - Type III]**

| **Source** | **Sum of Squares** | **df** | **Mean Square** | **F Value** | **p-value Prob > F** | |
|---|---|---|---|---|---|---|
| **Model** | 20.27 | 31 | 0.65 | 67.00 | < 0.0001 | **SIGNIFICANT** |
| **A-Temperature** | 0.51 | 1 | 0.51 | 52.56 | < 0.0001 | |
| **B-pH** | 3.55 | 1 | 3.55 | 364.13 | < 0.0001 | |
| **C-[Gal]** | 0.23 | 1 | 0.23 | 23.88 | < 0.0001 | |
| **D-Y.E** | 0.13 | 1 | 0.13 | 13.27 | 0.0009 | |
| **E-$(NH_4)_2SO_4$** | 1.03 | 1 | 1.03 | 105.40 | < 0.0001 | |
| **AB** | 0.06 | 1 | 0.06 | 5.75 | 0.0225 | |
| **AC** | 1.84 | 1 | 1.84 | 188.98 | < 0.0001 | |
| **AD** | 0.53 | 1 | 0.53 | 54.70 | < 0.0001 | |
| **AE** | 0.09 | 1 | 0.09 | 8.96 | 0.0053 | |
| **BC** | 0.02 | 1 | 0.02 | 2.55 | 0.1203 | |
| **BD** | 0.04 | 1 | 0.04 | 4.40 | 0.0440 | |
| **BE** | 1.14 | 1 | 1.14 | 117.11 | < 0.0001 | |
| **CD** | 0.86 | 1 | 0.86 | 88.02 | < 0.0001 | |
| **CE** | 0.07 | 1 | 0.07 | 7.44 | 0.0103 | |
| **DE** | 0.01 | 1 | 0.01 | 0.58 | 0.4523 | |
| **ABC** | 1.70 | 1 | 1.70 | 174.22 | < 0.0001 | |
| **ABD** | 0.45 | 1 | 0.45 | 46.19 | < 0.0001 | |
| **ABE** | 0.79 | 1 | 0.79 | 81.43 | < 0.0001 | |
| **ACD** | 0.74 | 1 | 0.74 | 75.71 | < 0.0001 | |
| **ACE** | 0.53 | 1 | 0.53 | 53.92 | < 0.0001 | |
| **ADE** | 1.54 | 1 | 1.54 | 157.47 | < 0.0001 | |
| **BCD** | 0.80 | 1 | 0.80 | 81.97 | < 0.0001 | |
| **BCE** | 0.12 | 1 | 0.12 | 12.45 | 0.0013 | |
| **BDE** | 0.00 | 1 | 0.00 | 0.41 | 0.5284 | |
| **CDE** | 1.27 | 1 | 1.27 | 130.03 | < 0.0001 | |
| **ABCD** | 0.12 | 1 | 0.12 | 11.87 | 0.0016 | |
| **ABCE** | 0.59 | 1 | 0.59 | 59.97 | < 0.0001 | |
| **ABDE** | 0.72 | 1 | 0.72 | 73.72 | < 0.0001 | |
| **ACDE** | 0.05 | 1 | 0.05 | 4.87 | 0.0346 | |
| **BCDE** | 0.72 | 1 | 0.72 | 73.81 | < 0.0001 | |
| **ABCDE** | 0.01 | 1 | 0.01 | 1.30 | 0.2632 | |

| | | | |
|---|---|---|---|
| **Std. Dev.** | 0.10 | **R-Squared** | 0.98 |
| **Mean** | 3.26 | **Adj R-Squared** | 0.97 |

| | | | |
|---|---|---|---|
| **C.V. %** | 3.03 | **Pred R-Squared** | 0.94 |

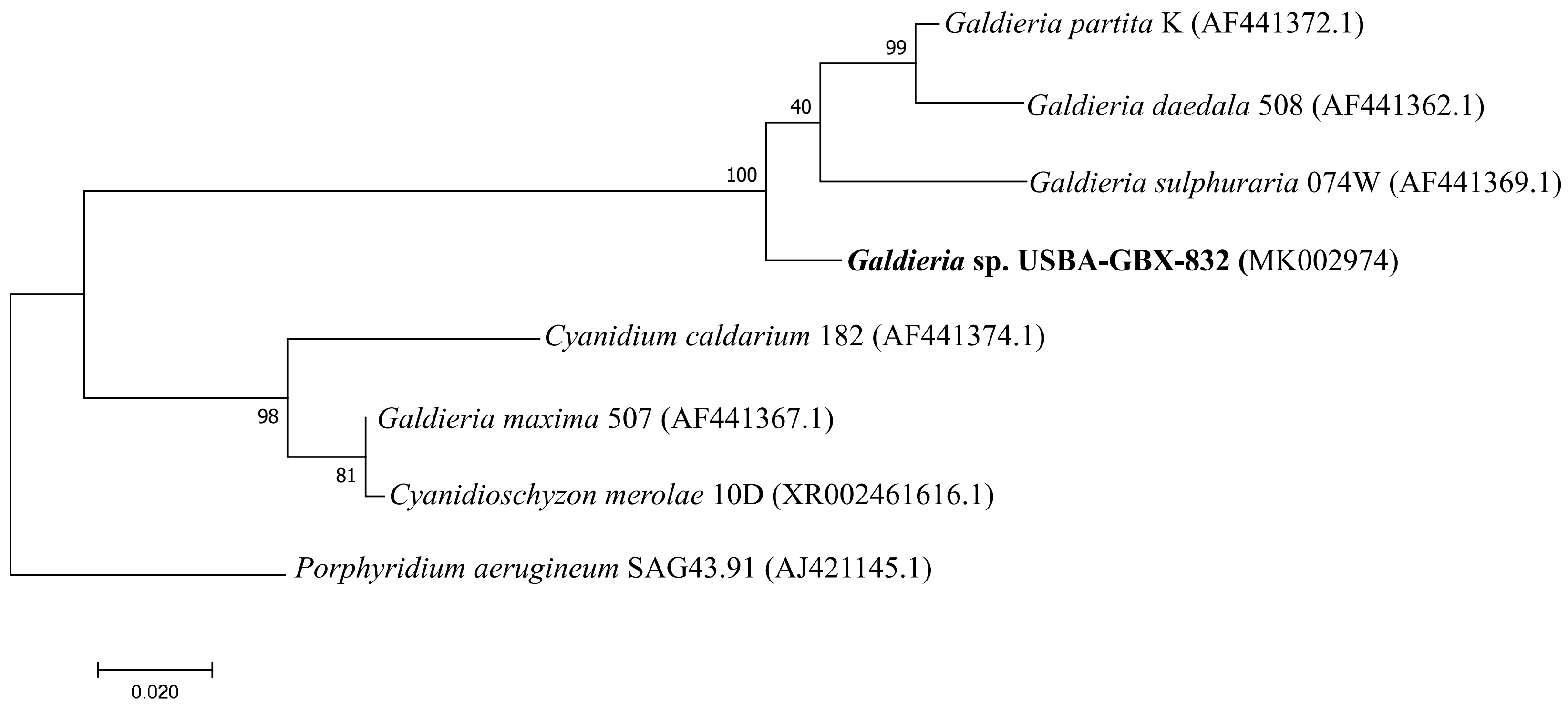


**Figure S1.** Phylogenetic tree of *Galdieria* sp. USBA-GBX-832 inferred from alignment of 18S rRNA gene and recognized type species of Family Cyanidiaceae. The tree was reconstructed by using the Maximum Likelihood method based on the Tamura-Nei model. Numbers represent percentages from 1000 replicate bootstrap sampling. Bar, 0.02 substitutions per nucleotide position. The microalgae *Porphyridium aerugineum* SAG43.91 (AJ421145.1) was used as outgroup.

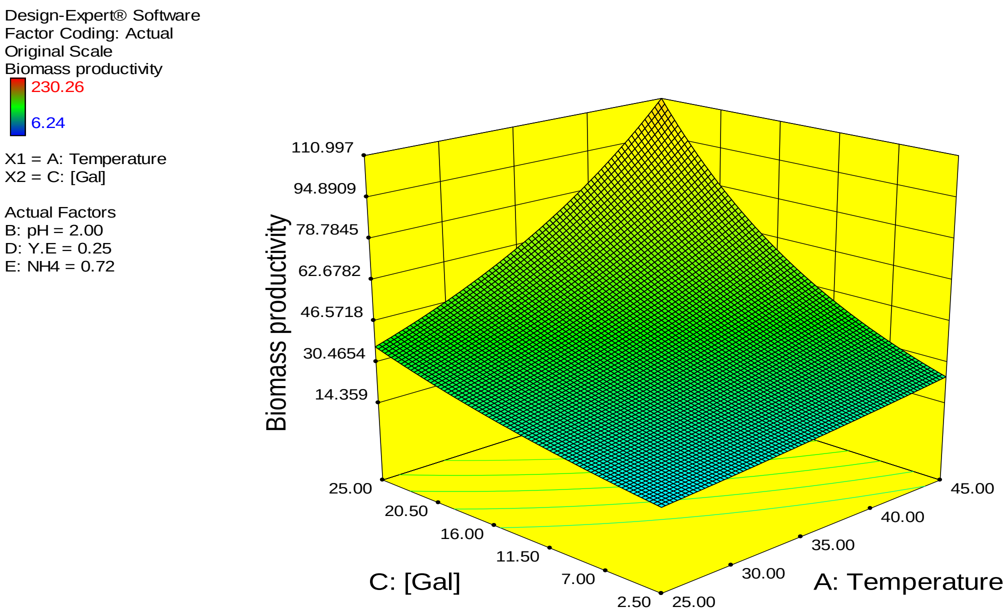


**Figure S2a.** Response surface curve showing the effect of carbon concentration and temperature over biomass productivity of *Galdieria* sp. USBA-GBX-832

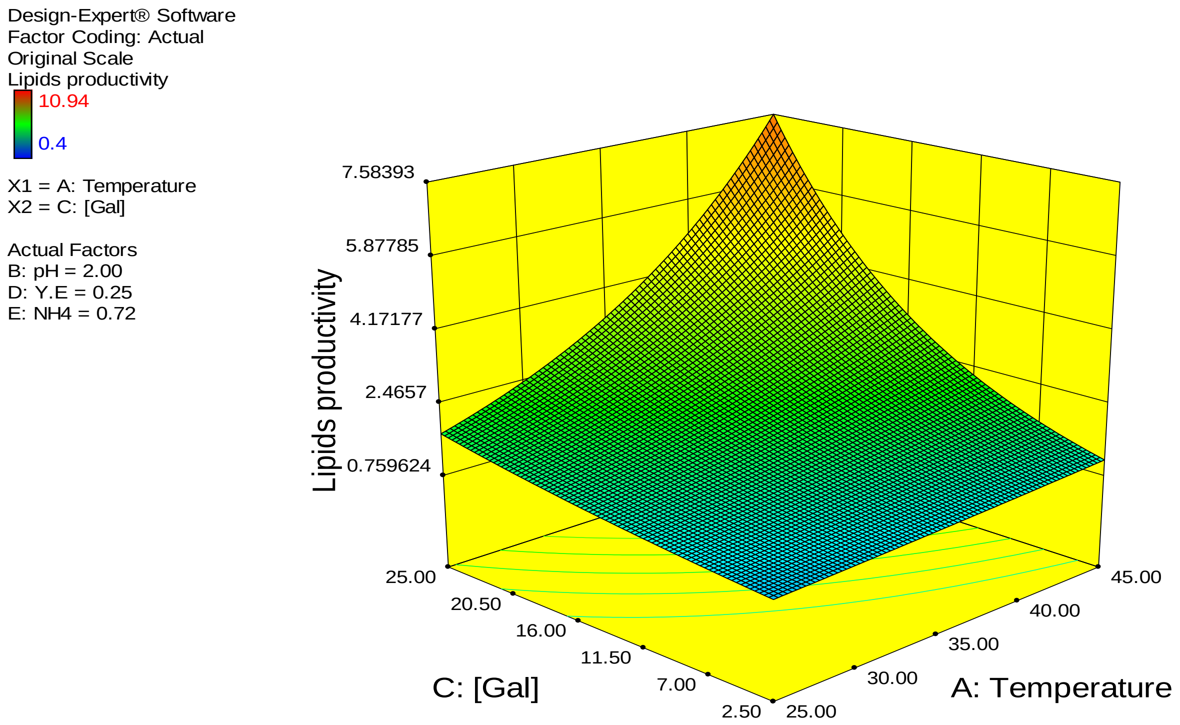


**Figure S2b.** Response surface curve showing the effect of carbon concentration and temperature over total lipid productivity of *Galdieria* sp. USBA-GBX-832

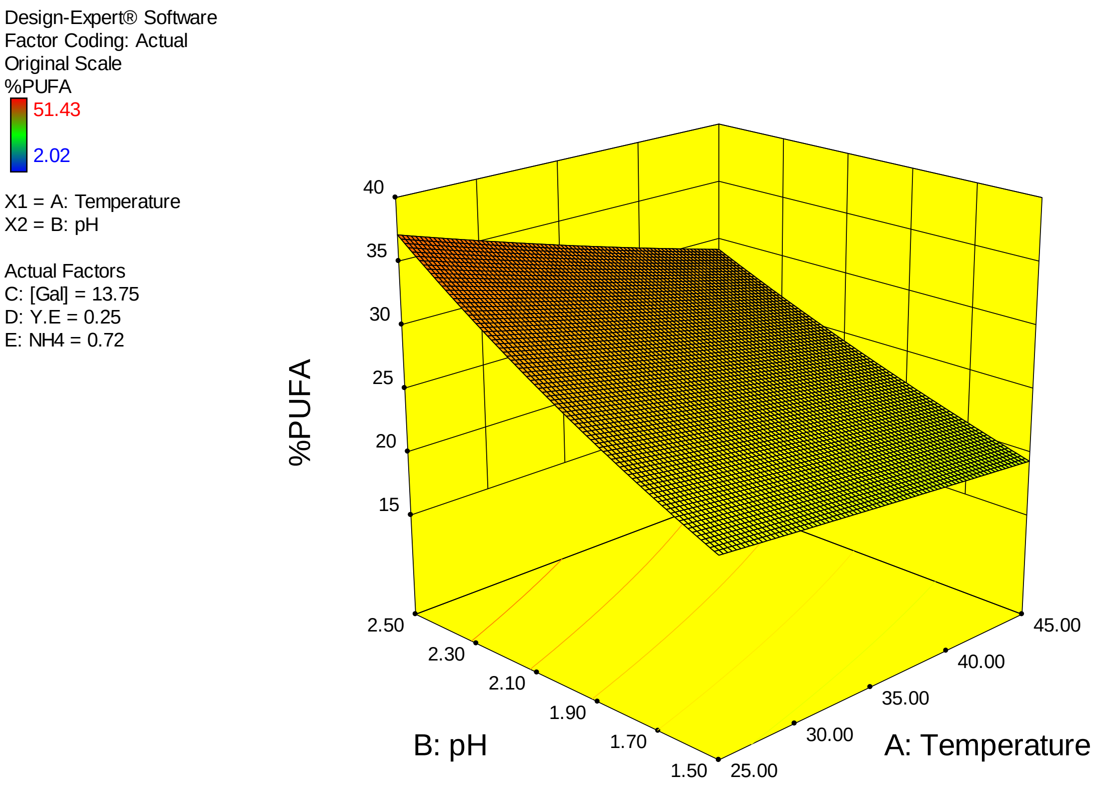


**Figure S2c**. Response surface curve showing the effect of pH and temperature over percentage of polyunsaturated fatty acid productivity (PUFA) of *Galdieria* sp. USBA-GBX-832

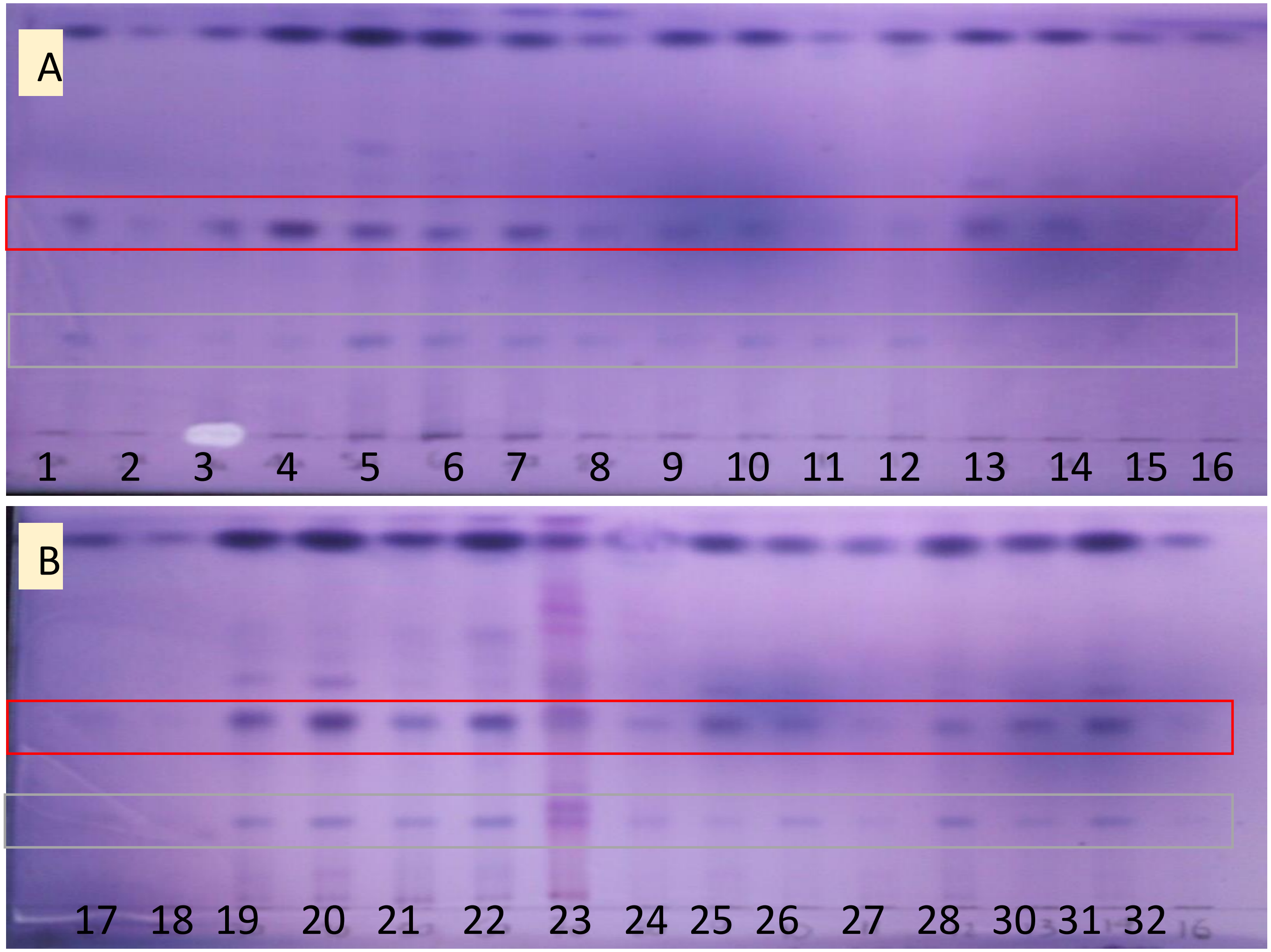


**Figure S3.** Evaluation of neutral lipids through TLC plates. A) treatments at 45 ° C and B) treatments at 25 ° C. Solvents hexane: ethyl acetate (7: 3). Revealed with *p*-anisaldehyde. The red boxes show the position of triacylglycerides ($C_{16:0}$) and the gray boxes the position of free fatty acids.
